# Heterogeneous Pd catalysts as emulsifiers in Pickering emulsions for integrated multistep synthesis in flow chemistry

**DOI:** 10.3762/bjoc.14.52

**Published:** 2018-03-19

**Authors:** Katharina Hiebler, Georg J Lichtenegger, Manuel C Maier, Eun Sung Park, Renie Gonzales-Groom, Bernard P Binks, Heidrun Gruber-Woelfler

**Affiliations:** 1 Institute of Process and Particle Engineering, Graz University of Technology, Inffeldgasse 13/III, 8010 Graz, Austria; 2 School of Mathematics and Physical Sciences, University of Hull, Hull HU6 7RX, UK

**Keywords:** compartmentalisation, heterogeneous catalysis, multistep flow chemistry, palladium, Pickering emulsions

## Abstract

Within the “compartmentalised smart factory” approach of the ONE-FLOW project the implementation of different catalysts in “compartments” provided by Pickering emulsions and their application in continuous flow is targeted. We present here the development of heterogeneous Pd catalysts that are ready to be used in combination with biocatalysts for catalytic cascade synthesis of active pharmaceutical ingredients (APIs). In particular, we focus on the application of the catalytic systems for Suzuki–Miyaura cross-coupling reactions, which is the key step in the synthesis of the targeted APIs valsartan and sacubitril. An immobilised enzyme will accomplish the final product formation via hydrolysis. In order to create a large interfacial area for the catalytic reactions and to keep the reagents separated until required, the catalyst particles are used to stabilise Pickering emulsions of oil and water. A set of Ce–Sn–Pd oxides with the molecular formula Ce_0.99−_*_x_*Sn*_x_*Pd_0.01_O_2−δ_ (*x* = 0–0.99) has been prepared utilising a simple single-step solution combustion method. The high applicability of the catalysts for different functional groups and their minimal leaching behaviour is demonstrated with various Suzuki–Miyaura cross-coupling reactions in batch as well as in continuous flow employing the so-called “plug & play reactor”. Finally, we demonstrate the use of these particles as the sole emulsifier of oil–water emulsions for a range of oils.

## Introduction

Palladium (Pd) catalysis has been established as a key component in the toolbox of organic chemists. Reactions that are catalysed by palladium benefit from the remarkable versatility and functional-group tolerance of the transition metal as well as the ability to control the reaction selectivity [1–3]. Pd catalysts have been implemented in the synthesis of various active pharmaceutical ingredients (APIs), natural products and agrochemicals amongst others [4]. In particular, Pd-catalysed C–C cross-coupling reactions have become indispensable in many modern synthetic protocols both in the laboratory and on an industrial scale. A highly important representative of this class of transformation is the Suzuki–Miyaura reaction [5–6], involving the coupling of aryl halides with phenylboronic acids yielding the corresponding biphenyls as product [7]. The biphenyl unit is a common structural motif in various pharmaceutically active agents and plays a crucial role in the binding affinity and the oral bioavailability of diverse APIs [8], including antihypertensive [9] and antitumour agents [10]. Advantages of the Suzuki–Miyaura coupling are mild reaction conditions, commercial availability of a large number of boronic acids and simple product purification [7]. Concerning the transition metal source for C–C cross-coupling reactions, homogeneous and heterogeneous Pd catalysts are utilised.

The employment of a variety of different ligands such as phosphines, amines and carbenes allows precise tuning of the properties of homogeneous Pd catalysts, which led to significant improvements in turn over number (TON), reaction rates, enantioselectivity as well as catalyst robustness and lifetime. Apart from that, ligand-free Pd catalysts are also known in the literature [11–12]. However, homogeneous Pd catalysis often requires catalyst loadings in the order of mol % to achieve effective coupling and suffers from catalyst re-use and recycling problems [11,13]. Furthermore, concerning the synthesis of pharmaceuticals, tedious purification steps need to be performed in order to remove residual metals. Considering these drawbacks of homogeneous catalysis, easily recoverable and recyclable heterogeneous Pd catalysts are much more attractive with respect to ecological and economical aspects [13]. One possibility to prepare heterogeneous transition metal catalysts is to immobilise palladium directly on a solid support such as activated carbon [14], zeolites [15], modified silica [16–18] or molecular sieves [19] to name but a few. Another option is the complexation of palladium by ligands which are covalently bound to the support material [12]. One example of such a catalyst was reported by our group using a bis(oxazoline) ligand bonded to 3-mercaptopropyl-functionalised silica [20]. Alternatively, the use of unsupported Pd nanoparticles or encapsulated Pd complexes are strategies to realise heterogeneous palladium catalysis [21]. Immobilisation of catalytic systems on solid supports can mitigate a lot of problems of homogeneous catalysts, for example, it allows a straightforward removal of the catalyst from the reaction system. However, most heterogeneous approaches require more drastic reaction conditions in comparison to their homogeneous counterparts, which often introduce undesirable leaching effects [17,20,22–23]. Consequently, the synthesis and application of unprecedented nonleaching heterogeneous palladium catalysts for cross-coupling reactions have been investigated intensively and vigorous efforts are made to implement them in industrial synthesis [11,13,20].

The idea of the so-called “compartmentalised smart factory” within the ONE-FLOW project [24] is to go a step further and combine different kinds of chemo- and biocatalysts in one “compartment". For this approach and to keep the reagents separated until required, the catalysts are contained within Pickering emulsions of oil and water phases. Emulsions are thermodynamically unstable mixtures of two immiscible liquids, e.g., oil and water, with typical droplet sizes in the micron range. Traditionally, emulsions have been kinetically stabilised by molecular species including surfactants, polymers or proteins all of which possess water-liking groups and oil-liking groups enabling them to adsorb to freshly created oil–water interfaces preventing to some extent coalescence between neighbouring droplets [25]. So-called Pickering emulsions, however, are stabilised solely by solid colloidal particles which can adsorb to droplet interfaces forming a protective layer endowing the emulsion with extremely high stability to coalescence [26]. Examples of suitable particles include silica, alumina, metals, polymers and proteins of different sizes and shapes. One of the key factors influencing the effectiveness of a particle to act as an emulsifier is its wettability normally quantified by the contact angle θ the particle makes with the oil–water interface (through water). For relatively hydrophilic particles, θ < 90° and preferred emulsions are oil-in-water (o/w). For relatively hydrophobic particles, θ > 90° and water-in-oil (w/o) emulsions are preferred [27]. Particles of intermediate wettability are well held at a fluid interface as the energy required to remove them can be several thousand *kT* (*k* is Boltzmann constant, *T* is temperature); such particles are deemed irreversibly adsorbed under quiescent conditions. As summarised recently [28], the two liquids may be oil and water, two immiscible oils or even two immiscible water phases and different particles need to be designed in each case to impart stabilisation of dispersed drops in a continuous phase. In 2010, Crossley et al. [29] put forward the idea that catalyst particles may both act simultaneously as an emulsifier in Pickering emulsions and serve as the catalyst in which water-soluble reactants and oil-soluble reactants react at the oil–water interface populated by catalyst particles. They deposited metallic Pd onto carbon nanotube–inorganic oxide hybrid nanoparticles and used them in emulsions for the hydrodeoxygenation of a phenolic compound and the hydrogenation and etherification of an aldehyde. The advantages of such a system include a high interfacial area for reaction, the ultrastability of emulsion drops during reaction and easy recovery of the catalyst particles and products as emulsions may be rendered unstable subsequently. A mini-review of the area of Pickering emulsion interfacial catalysis appeared in 2015 [30].

**Figure 1:**
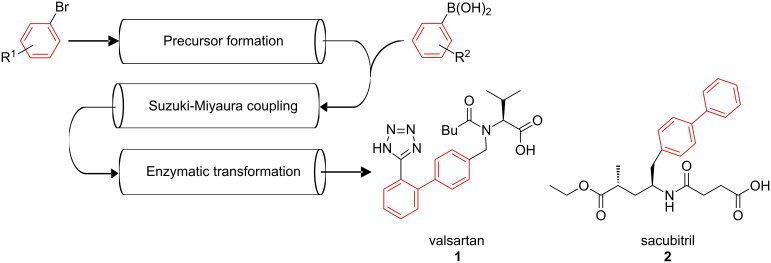
Targeted integrated multistep synthesis of valsartan (**1**) and sacubitril (**2**).

In this work an outlook on the planned realisation of the integrated multistep continuous flow synthesis of valsartan and sacubitril within the frame of the ONE-FLOW project is given. The compounds are well known as APIs in a combination drug for the treatment of hypertension and chronic heart failure (Entresto^®^, Novartis) [31–35]. A preliminary scheme of the planned synthetic route is shown in Figure 1. As can be seen, the key step of our processes is the formation of the biaryl unit via a Suzuki–Miyaura cross-coupling reaction. To provide solid Pd catalysts with a high potential for the planned approaches, a set of Ce–Sn–Pd oxides with the molecular formula Ce_0.99−_*_x_*Sn*_x_*Pd_0.01_O_2−δ_ (*x* = 0–0.99; δ indicates the oxygen vacancies in the crystal lattice of the oxides, the values for delta are rather small, thus the value for oxygen is ≈2, [36–37]) is tested for that approach. The tolerance of the catalysts towards different functional groups and their minimal leaching which has already been demonstrated with various Suzuki–Miyaura cross-coupling reactions in batch as well as in continuous flow employing the so-called plug & play reactor [38], is summarised. In this work we present for the first time the use of these particles as the sole emulsifier of oil–water emulsions for a range of oils.

## Results and Discussion

### Metal oxide-supported ionic palladium catalysts

#### Synthesis

The heterogeneous Ce–Sn–Pd compounds were synthesised using the solution combustion technique according to Baidya et al. [39] with slight modifications [37]. The benefits of this single-step method are the simplicity of the procedure, the usage of nontoxic and inexpensive precursors and that several grams of material can be obtained within hours. To study the influence of the amounts of Ce and Sn on the catalytic behaviour, five mixed oxides with the molecular formula Ce_0.99−_*_x_*Sn*_x_*Pd_0.01_O_2−δ_ (*x* = 0, 0.20, 0.495, 0.79 and 0.99) were synthesised and attained in quantitative amounts (>99%). The obtained solids can be used directly for characterisation as well as for the activity tests.

#### Characterisation

The Ce–Sn–Pd catalysts were analysed using different state-of-the-art methods. Details of the characterisation were published recently [37]. Here, only the most important characteristics are summarised. The obtained X-ray diffraction (XRD) profiles proved the nanocrystallinity of the catalyst particles, which either show single phase cubic, tetragonal or a more amorphous cubic/tetragonal mixed phase structure depending on the content of Sn. As far as palladium substitution in the lattice is concerned, XRD and X-ray photoelectron spectroscopy (XPS) analysis approved the predominant cationic nature of incorporated palladium (Pd^2+^) and revealed only minor amounts of metallic Pd. Brunauer–Emmett–Teller (BET) measurements showed that the specific surface areas of the five catalysts range from 27–98 m^2^/g. Particle sizes of ≈10–100 µm were measured with a monomodal size distribution for the cerium-rich catalysts and a polymodal distribution for catalysts rich in tin.

### Suzuki–Miyaura coupling reactions

#### Batch reactions

**Scheme 1:**
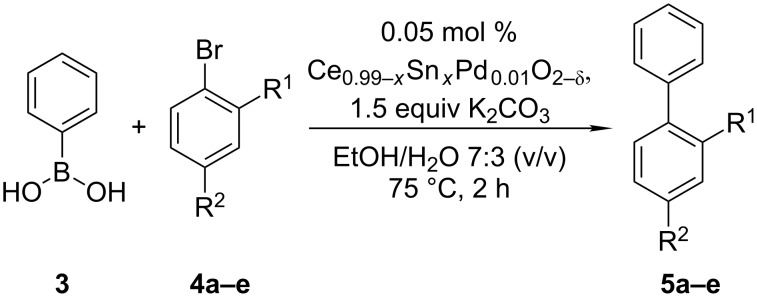
Suzuki–Miyaura coupling of phenylboronic acid **3** with various bromoarenes **4a–e** (**a**: R^1^ = H, R^2^ = CH_3; _**b**: R^1^ = H, R^2^ = OH; **c**: R^1^ = H, R^2^ = COOCH_3; _**d**: R^1^ = H, R^2^ = CF_3; _**e**: R^1^ = CN, R^2^ = H).

The catalytic activity of the synthesised catalysts in the Suzuki–Miyaura reaction was investigated using phenylboronic acid **3** in combination with various bromoarenes **4a–e**, featuring *ortho*- and *para*-substitution of electron donating as well as electron withdrawing functional groups, as coupling partners (Scheme 1). Concerning the targeted synthesis of **1**, aryl halide **4e** was of special interest as the cyano group is known to be convertible to the *ortho*-tetrazole moiety [9] present in the API. Based on prior optimisation studies [37], reactions were performed in EtOH/H_2_O 7:3 (v/v) at 75 °C using K_2_CO_3_ as inorganic base. Phenylboronic acid as well as K_2_CO_3_ were added in 50% molar excess relative to the corresponding aryl bromide, whereas a catalyst amount corresponding to 0.05 mol % of Pd was used. The reaction progress was monitored by high-performance liquid chromatography (HPLC).

In general, the catalysts with tin proportions of 0.20, 0.79 and 0.99 proved to be most effective in the desired transformations with exceptional high activities (turn over frequency, TOF > 12,000 h^−1^) in all tested Suzuki–Miyaura cross-coupling reactions. While *para*-substituted bromoarenes were converted quantitatively within a reaction time of 2 h with an order of 4-bromoacetophenone (**4c**) ≥ 4-bromobenzotrifluoride (**4d**) > 4-bromotoluene (**4a**) > 4-bromophenol (**4b**), the coupling of 2-bromobenzonitrile (**4e**) required the 4-fold amount of catalyst to reach full conversion. *p*-Bromobenzenes containing electron-withdrawing substituents showed higher reactivity than bromobenzenes containing electron-donating groups. When Ce_0.495_Sn_0.495_Pd_0.01_O_2–δ_ was employed as catalyst, however, the catalytic activity was found to be significantly lower and the binary oxide Ce_0.99_Pd_0.01_O_2–δ_ showed to be least efficient for the explored Suzuki–Miyaura couplings. Regarding the selectivity of the catalysts in the selected cross-coupling reactions, no bromoarene-deriving side products (dehalogenation product Ar’ and bromoarene homocoupling product Ar’Ar’) could be detected. However, both boronic acid homocoupling (ArAr) and boronic acid oxidation (ArOH) occurred to a small extent as indicated by HPLC. As the side product formation mainly occurs when the bromoarene coupling partner gets depleted, highest reaction selectivity (up to 99.5% [37]) can be achieved by termination of the transformation just at the moment of full conversion.

In conclusion, Ce_0.99−_*_x_*Sn*_x_*Pd_0.01_O_2−δ_ (*x* = 0–0.99) proved to be very active catalysts for Suzuki–Miyaura reactions. Based on the experimental results, the tin and cerium content of the catalysts respectively do not seem to be directly connected to their catalytic activity. Nevertheless, there are indications that the surface area and the particle size distribution play a role in the catalytic performance of the palladium substituted metal oxides. In view of the aimed application of the Pd metal oxides for targeted API synthesis, the obtained results are very promising although the relevant transformation needed higher catalyst amounts.

#### Recyclability and metal leaching

In the context of heterogeneous catalysis it is essential to investigate the actual nature of the catalysts during the reaction as well as the reusability and stability of the catalytic active compounds. To assess the reusability of the heterogeneous catalysts, the Pd-substituted metal oxides were subjected to Suzuki–Miyaura couplings of 4-bromotoluene with phenylboronic acid in five subsequent reactions, i.e., after one reaction was finished, the particles were separated from the solution via filtration, washed, dried and reused for a new reaction with new substrates. Although during the filtration and drying steps catalyst loss could not be prevented, a high degree of recyclability was established and only a minor decrease of mass specific catalyst activity was observed [37].

As far as palladium leaching is concerned, ICP–MS measurements revealed 0.06–0.14 mg/L Pd in the reaction solution after the 3rd reaction run [37]. This finding is in accordance with the theory that the actual catalyst of the coupling reaction is leached palladium and supports a reaction mechanism via homogeneous catalysis. For the purpose of elucidating the homo- or heterogeneous nature of synthesised catalysts in more detail, further studies including a hot filtration test and catalyst poisoning [40–41] were performed [37]. In summary, the obtained results confirm a palladium release and capture mechanism. The synthesised heterogeneous metal oxides act as pre-catalysts, which slowly release minimal amounts of active palladium into solution. However, as levels of leached Pd are below the regulatory limits for orally administered pharmaceuticals [41], the mixed metal oxides have high potential as heterogeneous catalysts for the synthesis of APIs, such as **1** and **2**.

#### Suzuki–Miyaura reactions in continuous flow

After the applicability and versatility of the palladium substituted cerium tin oxides was confirmed in batch, the aim was to implement the catalysts in a continuous flow setup, which is also suitable for the multistep synthesis of **1** and **2**. Therefore, the so-called plug & play reactor, a versatile device featuring both exchangeable reaction segments and modules for heating/cooling and mixing [38], was employed to test the activity and stability of the catalysts in continuous flow. In the plug & play reactor the reaction media flow through 1 mm tubes embedded in channels filled with heating/cooling media, ensuring both, enhanced mixing and rapid heat transfer. Commercially available HPLC columns filled with catalyst particles serve as fixed-bed reactors and contribute to the high versatility of the device**.** With this approach, gas–solid, liquid–solid as well as gas–liquid–solid reactions can be realised within the upper performance limits of 200 °C and 40 bar [38].

The performance of the plug & play reactor in terms of continuous Suzuki–Miyaura cross coupling was investigated using phenylboronic acid (**3**) in combination with different *ortho*- and *para*-substituted bromoarenes in aqueous ethanolic mixtures employing K_2_CO_3_ as base [38]. Monitoring of the reaction progress via inline UV–vis spectroscopy as well as offline HPLC analysis [38] led to the result that after an initial induction phase (≈30 min) a stable process was achieved, forming the respective products in high yields (up to 99%) for more than 30 h with excellent selectivity without catalyst deactivation [42]. As expected, increasing the catalyst amount and residence time by use of three sequential fixed bed reactors enhanced product formation and conversions >90% were obtained. Furthermore, analysis of the crystalline product by means of ICP–MS confirmed only trace amounts of leached cerium (≈1 mg/kg final product), tin (≈0.2 mg/kg product) and palladium (≈1 mg/kg product). In addition to that, direct product isolation via integrated crystallisation was successfully implemented as a continuous downstream protocol [42].

Concluding, the plug & play reactor is a highly versatile device, which is applicable for different kinds of heterogeneously catalysed reactions. The results obtained strongly indicate the high potential for realising other reactions of interest to produce pharmaceutical and fine chemical intermediates in continuous flow, including the multistep synthesis of valsartan (**1**) and sacubitril (**2**). In addition to the approach with the compartmentalised catalysts in Pickering emulsions and thus the usage of one HPLC column filled with catalytic active compounds, the setup is optimal to employ multiple columns filled with different types of catalysts. Furthermore, the implementation of other functionalised materials, e.g., solid-supported scavengers for leached metals, is straightforward.

#### Size reduction of catalyst particles

**Figure 2:**
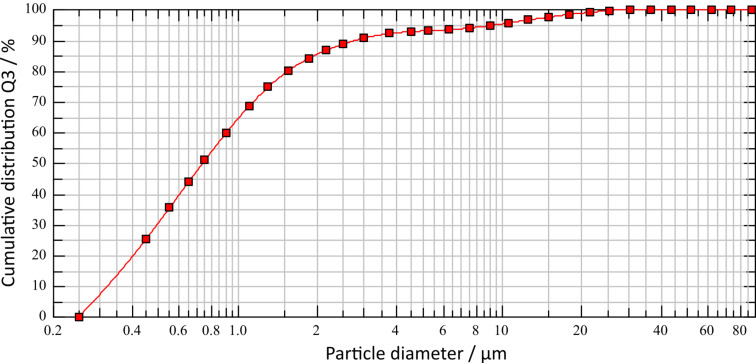
Particle size distribution of Ce_0.495_Sn_0.495_Pd_0.01_O_2–δ_ after size reduction via milling and separation via sedimentation in water.

As mentioned before, the as synthesised catalyst particles have particle diameters in the range of 10–100 µm. Since the stabilisation of micron-sized Pickering emulsion droplets requires particle sizes <1 μm, a decrease of particle size was necessary. For that purpose, a Resch PM10 planet ball mill equipped with metal balls (*d* = 8 mm, 500 rpm, 10 min milling time) was used for dry milling of powdered Ce_0.495_Sn_0.495_Pd_0.01_O_2–δ_ and wet milling (suspension in water) of Ce_0.20_Sn_0.79_Pd_0.01_O_2–δ_, respectively. These two catalysts were chosen since they showed the highest activities for Suzuki–Miyaura couplings in batch [37] and continuous flow [38]. After milling, the coarse and fine fractions of the particles were separated via sedimentation. For this purpose, 2 g of catalyst were suspended in 1 L water, the particles were de-aggregated by ultrasonic treatment and left to sediment. After a sedimentation time of 35 min, the upper half of the suspension was removed with a pipette and fractions were dried in a muffle furnace (120 °C until dryness, then at 350 °C overnight). Table 1 and Figure 2 indicate that for both catalysts 90% of the particles in the separated fines fractions are <5 µm (= *x*_90_) in diameter. The overall yield for this particle fraction was 20%, but future work will concentrate on the optimisation of this step. Microscopic pictures indicate spherical particle shape. The density of the particles, also important for the usage of the particles as stabilisers in Pickering emulsions, was determined to be 5.97 g/cm^3^ for Ce_0.495_Sn_0.495_Pd_0.01_O_2–δ_ and 5.38 g/cm^3^ for Ce_0.20_Sn_0.79_Pd_0.01_O_2–δ_.

**Table 1:** *x*_90_ and specific surface area of the catalysts after milling and separation *via* sedimentation.

Catalyst	*x*_90_ [µm]	BET surface area [m^2^ g^−1^]

Ce_0.495_Sn_0.495_Pd_0.01_O_2–δ_	2.84	28
Ce_0.20_Sn_0.79_Pd_0.01_O_2–δ_	4.20	58

#### Implementation of the catalyst particles in Pickering emulsions

For simplicity, we have investigated emulsions containing equal volumes of water (Milli-Q) and oil with catalyst particles first dispersed in water using an ultrasonic probe (Jencons Vibra-Cell, 5 min, 130 W). The oils chosen are the aliphatic alkane octane (Sigma, >99%, density 0.699 g/cm^3^), the cyclic alkane cyclohexane (Fisher Scientific, >99%, density 0.774 g/cm^3^) and the aromatic oil toluene (VWR Chemicals, >99%, density 0.865 g/cm^3^). Emulsions were prepared using a rotor-stator homogeniser (IKA T25 digital Ultra-Turrax) with a stator diameter of 8 mm for 1 min at 10,000 rpm at room temperature. Emulsion type was established using the drop test (addition of a drop of emulsion to either water or oil). The stability of emulsions to gravity-induced creaming or coalescence was assessed by monitoring the position of the water–emulsion or oil–emulsion interfaces respectively. Microscopy images of the emulsions were recorded using an Olympus BX53 microscope with GXCAM-U318 camera attached and GXCapture-T software.

#### Catalyst Ce_0.495_Sn_0.495_Pd_0.01_O_2–δ_

**Figure 3:**
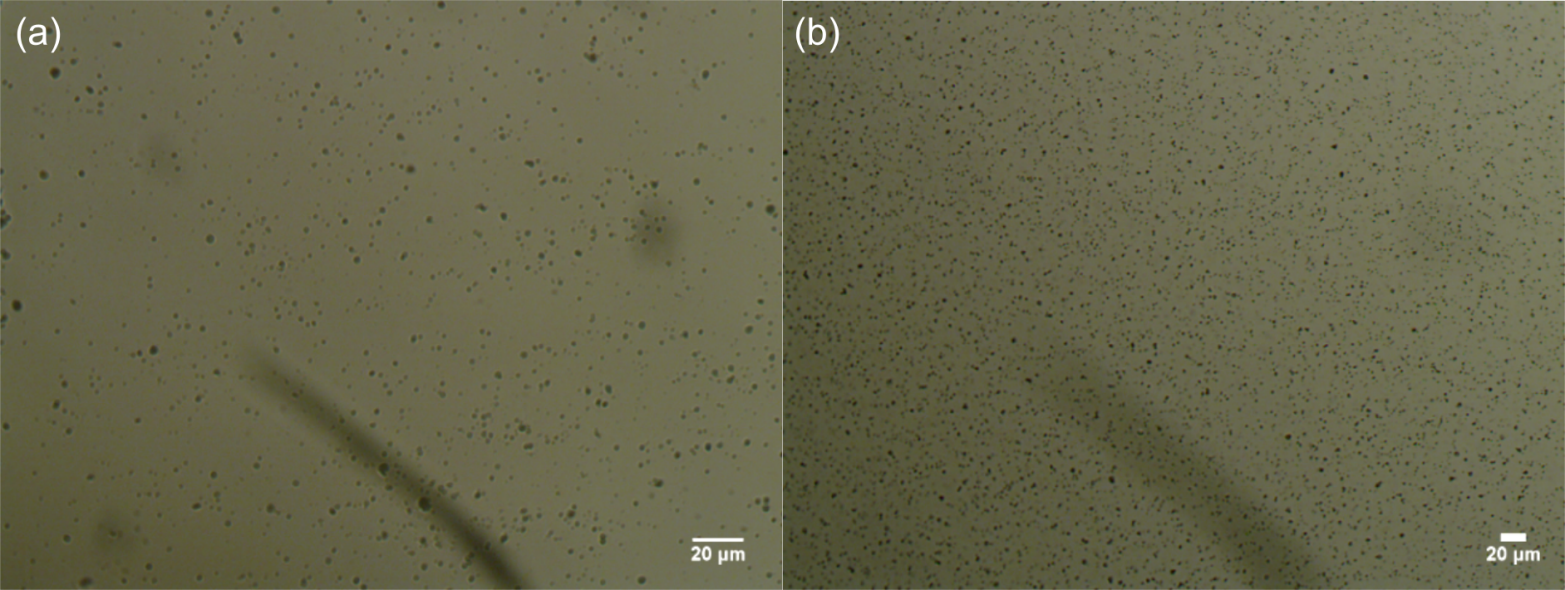
Optical microscope images of fresh aqueous dispersions, 0.05 wt %, of (a) Ce_0.495_Sn_0.495_Pd_0.01_O_2–δ_ and (b) Ce_0.20_Sn_0.79_Pd_0.01_O_2–δ_ particles.

Being relatively hydrophilic mixed oxides, both types of catalysts formed in water suspensions with discrete particles, at least at low concentration (0.05 wt %) (see Figure 3).

**Figure 4:**
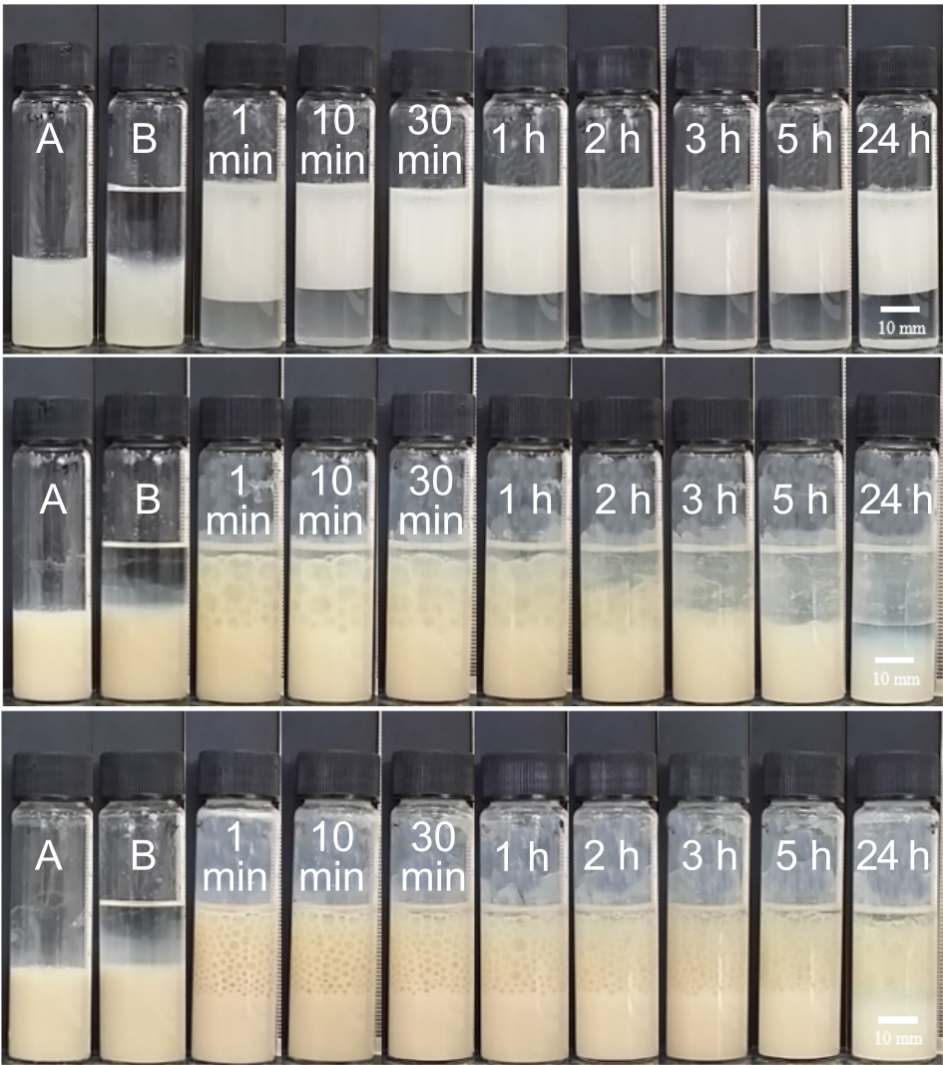
Photos of vessels containing cyclohexane-in-water emulsions stabilised by particles of Ce_0.495_Sn_0.495_Pd_0.01_O_2–δ_ at concentrations in water of 0.03 wt % (upper), 0.30 wt % (middle) and 1.0 wt % (lower) at different times since preparation. A is the aqueous dispersion of particles, B is after addition of oil but before emulsification.

Emulsions of all the oils are o/w in which their stability to both creaming and coalescence as well as their average droplet size depend markedly on particle concentration. As an example, the appearance of cyclohexane-in-water emulsions with time for three selected particle concentrations can be seen in Figure 4. This pattern of behaviour is followed with the other two oils also. For particle concentrations in water between 0.01 and 0.10 wt % (upper in Figure 4), reasonably stable emulsions to coalescence are formed although they exhibit creaming with the separation of a lower serum from which nonadsorbed particles sediment. Although the initial oil volume fraction in emulsions is 0.5 (*t* = 0), that in creamed emulsions after 1 day reaches as high as 0.7–0.8. Such excellent stability to coalescence in high internal phase emulsions is due to the particle layer on droplet interfaces acting as a barrier to drop fusion. Between 0.20 and 0.50 wt % (middle), although an emulsion forms initially, it rapidly coalesces until complete phase separation in some cases. At and above 1.0 wt % (lower), emulsions can be re-stabilised to some extent although visible oil drops (mm-sized) develop within the cream.

**Figure 5:**
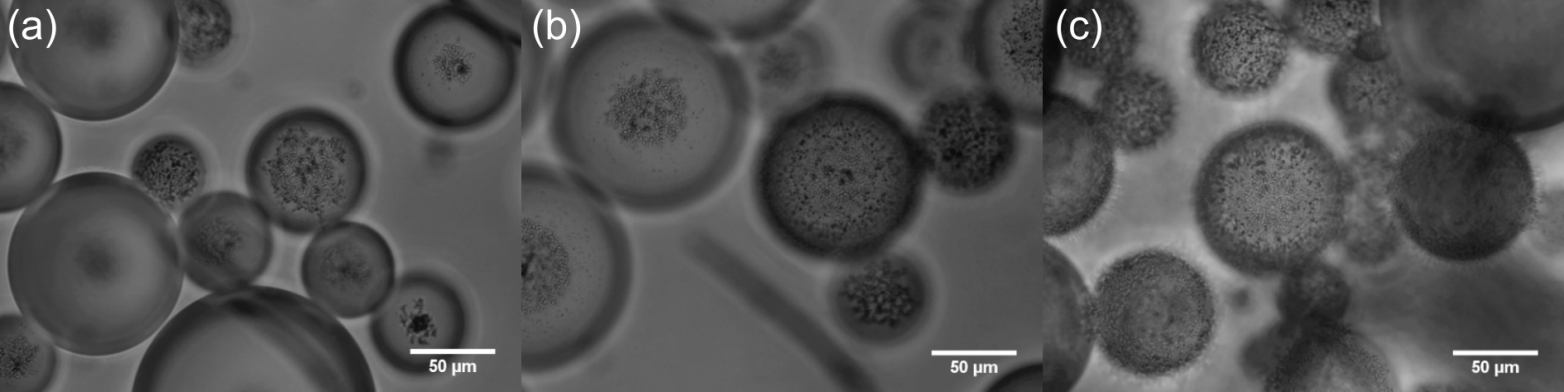
Optical microscopy images of cyclohexane-in-water emulsions of Figure 4 after one month for particle concentrations of (a) 0.02 wt %, (b) 0.05 wt % and (c) 0.10 wt %.

**Figure 6:**
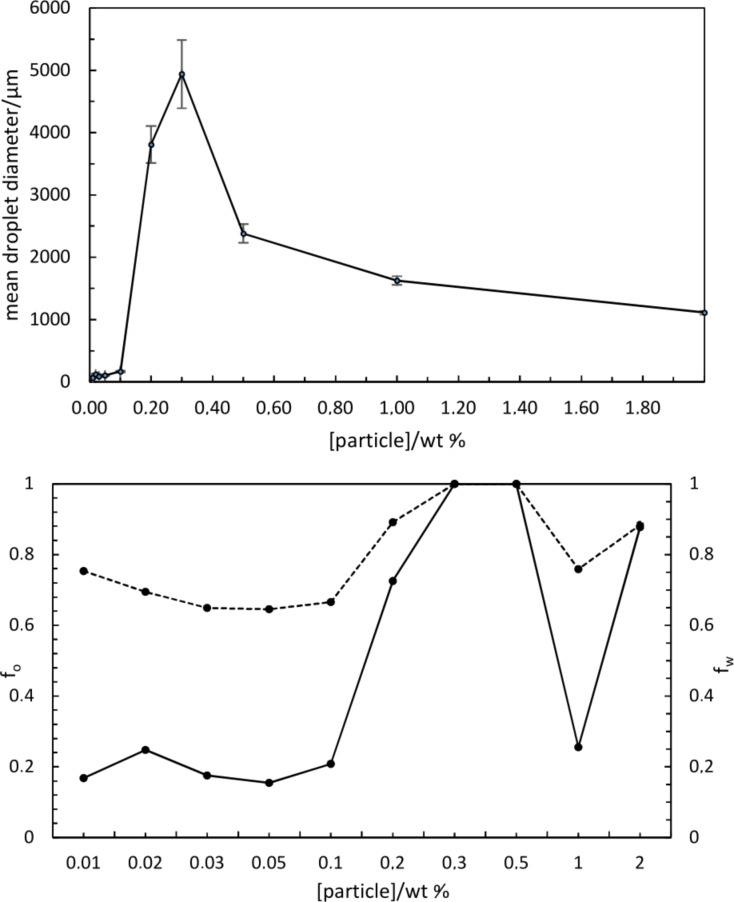
(top) Mean emulsion droplet diameter after 30 min as a function of particle concentration for system in Figure 4; (bottom) variation of f_o_ (solid line) and f_w_ (dashed line) after one month with particle concentration for emulsions in Figure 4 (note nonlinear scale).

**Figure 7:**
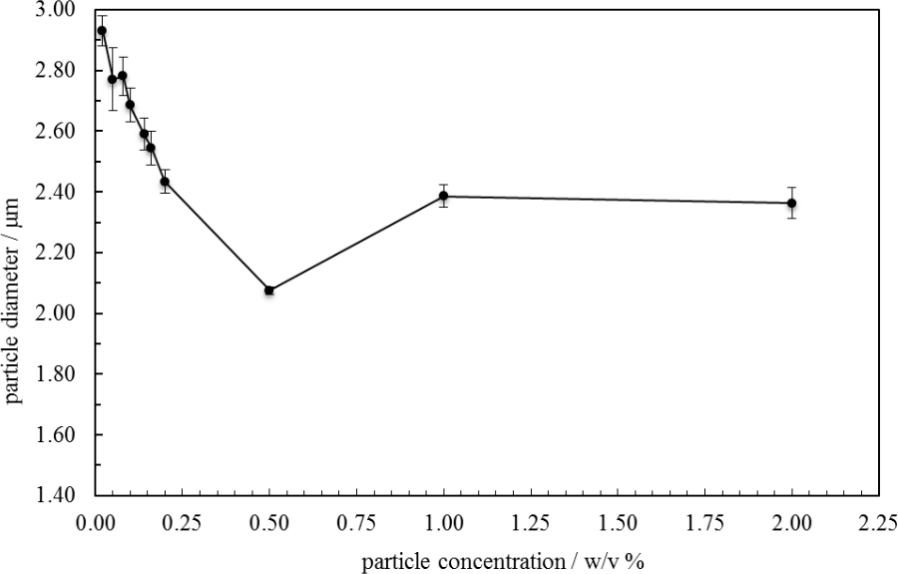
Mean particle diameter in aqueous dispersions as a function of Ce_0.495_Sn_0.495_Pd_0.01_O_2–δ_ concentration. Particle size measurements were carried out with a Mastersizer 2000 laser diffraction granulometer with the dispersion unit Hydro2000SM(A).

**Figure 8:**
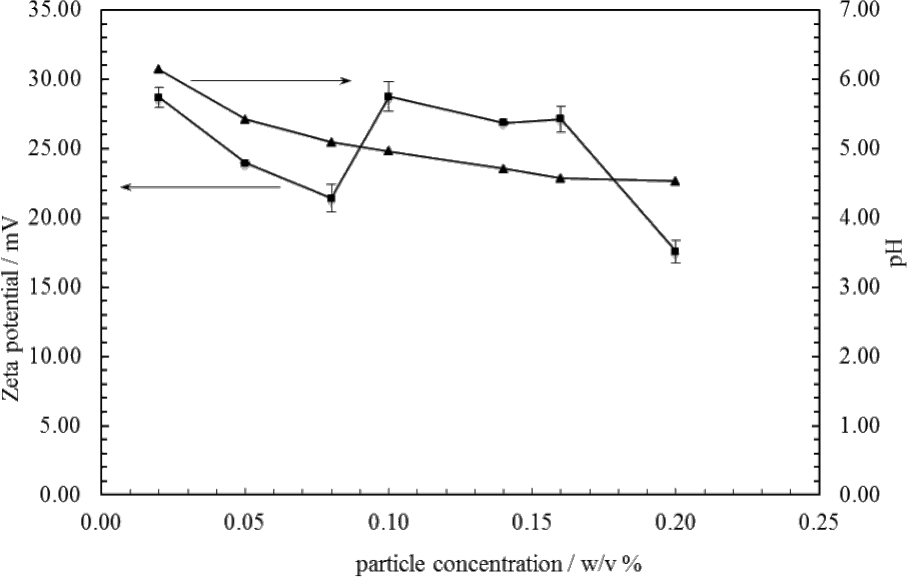
Variation of the zeta potential and pH value of aqueous dispersions of Ce_0.495_Sn_0.495_Pd_0.01_O_2–δ_ particles versus particle concentration. Zeta potentials were measured using the Malvern Zetasizer Nanoseries. pH values were determined at room temperature using a Jenway 3510 pH meter.

For the stable emulsions at low particle concentrations, optical microscopy reveals the presence of catalyst particles around oil droplets in water, Figure 5. Roughly, an increase in particle concentration results in increased coverage of droplets by particles. Since around 50% of the particles are sub-micron in size, however, a fraction of droplet interfaces may be coated in these particles which are of a size smaller than the resolution limit of the microscope. By contrast, the larger sized particles appear aggregated at droplet interfaces. The variation in the average oil droplet diameter with particle concentration is given in Figure 6 (top). Relatively small drops (between 70 and 150 μm) exist below 0.1 wt %, drops as large as 5 mm exist at 0.3 wt % after which the drop size decreases progressively to around 1 mm at 2 wt %. For emulsion stability with respect to coalescence, we define a parameter f_o_ = (vol. free oil at time *t*/vol. oil initially). Likewise, the stability to creaming is given by f_w_ = (vol. free water at time *t*/vol. water initially). Values of both parameters can vary from 0 (completely stable) to 1 (complete phase separation). After one month, the variation of f_o_ and f_w_ with particle concentration is given in Figure 6 (bottom). Up to 0.1 wt %, creaming is extensive and although some coalescence ensues, the residual emulsions remain stable for at least six months. Coalescence is then very extensive at 0.2, 0.3 and 0.5 wt % and mm-sized drops are formed in the early stages. Our first hypothesis was that the sudden increase in emulsion instability at higher particle concentrations may be due to particle aggregation in water prior to emulsification. Such large particle aggregates of high density would be weakly retained at the oil–water interfaces offering little protection to the coalescence between drops. However, a more detailed look at the particle size in water at different particle concentrations (Figure 7) as well as the unexpected results of the zeta potential and the pH value at different particle concentrations (Figure 8) are an indication that other effects, such as possible reactions of the particles with water, are the reason for the instability of the emulsions at higher concentrations.

**Figure 9:**
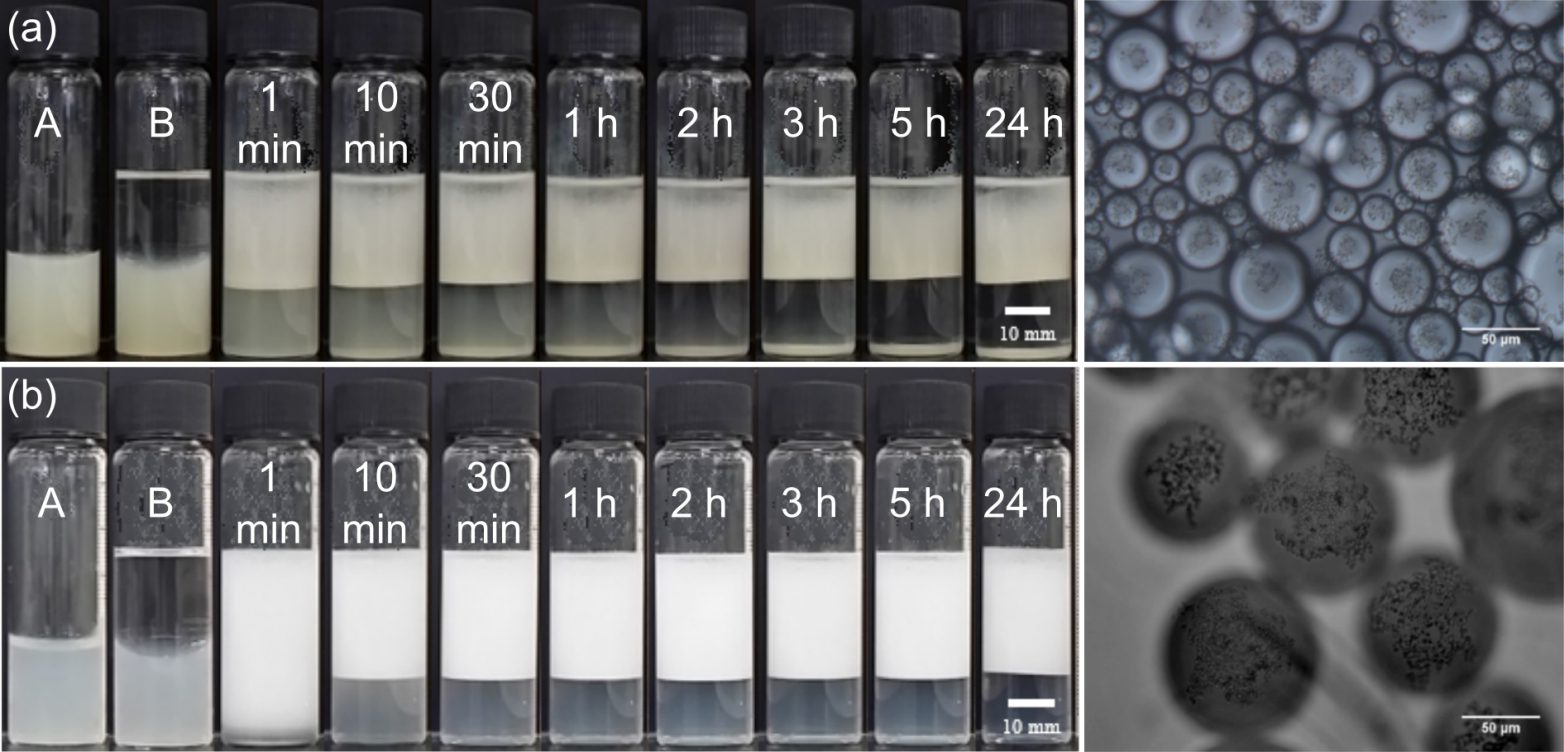
(a) Appearance of octane-in-water emulsions with time at 0.05 wt % of Ce_0.495_Sn_0.495_Pd_0.01_O_2–δ_ (left) and optical microscope image after 1 week (right); (b) Appearance of toluene-in-water emulsions with time at 0.01 wt % Ce_0.495_Sn_0.495_Pd_0.01_O_2–δ_ (left) and optical microscope image after 1 week (right).

Similar trends were found with octane and toluene as the oil phase with the most stable emulsions to coalescence and creaming appearing around ≤0.05 wt % particles (Figure 9).

#### Catalyst Ce_0.20_Sn_0.79_Pd_0.01_O_2–δ_

**Figure 10:**
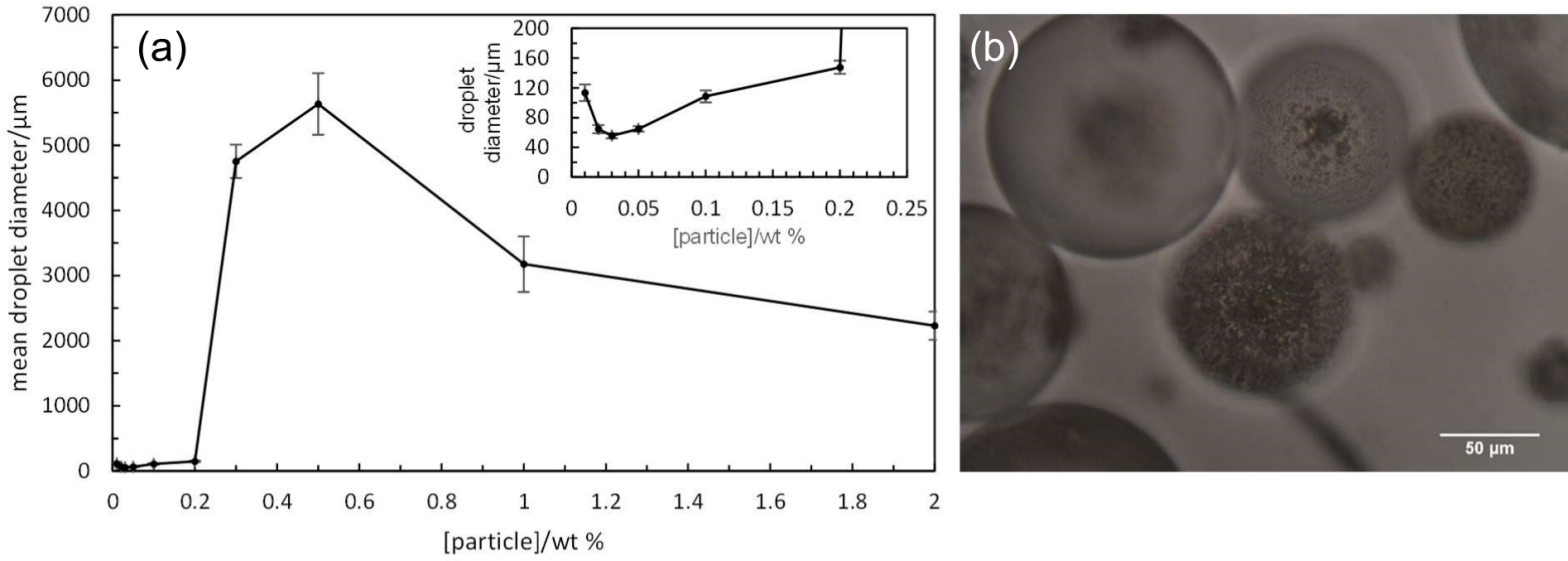
(a) Variation of droplet diameter with particle concentration for octane-in-water emulsions stabilised by Ce_0.20_Sn_0.79_Pd_0.01_O_2–δ_ particles after one month, (b) optical microscope image of the emulsion at 0.2 wt % particles.

**Figure 11:**
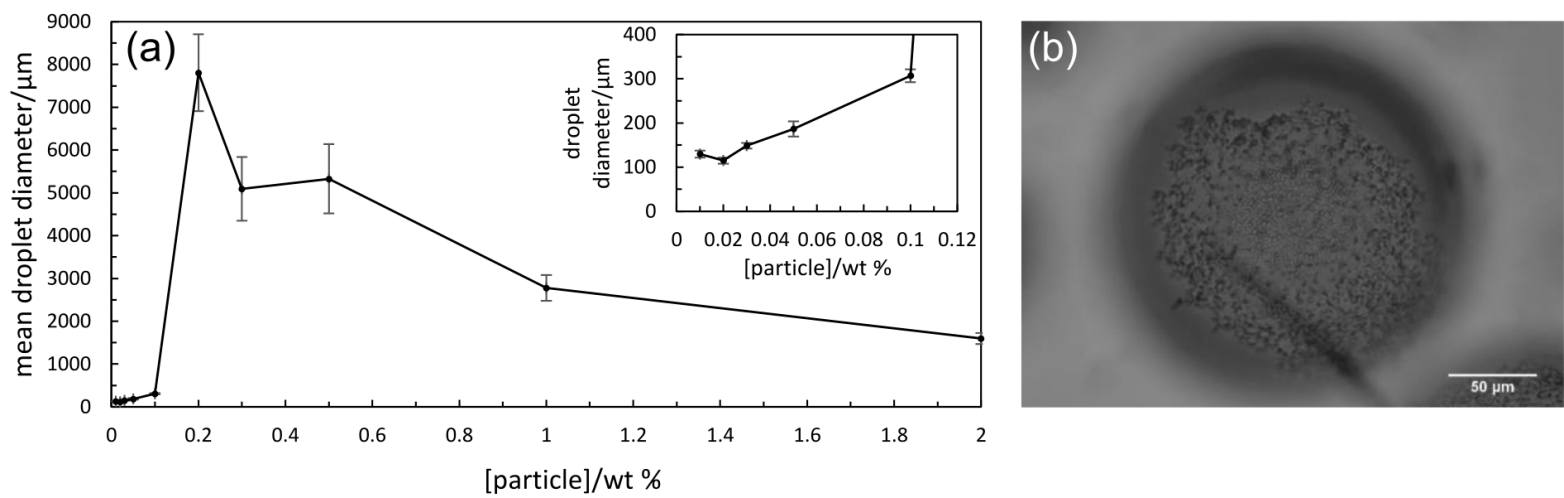
(a) Variation of droplet diameter with particle concentration for toluene-in-water emulsions stabilised by Ce_0.20_Sn_0.79_Pd_0.01_O_2–δ_ particles after one month, (b) optical microscope image of the emulsion at 0.1 wt % particles.

For these particles of larger mean size and with a higher Sn content, surprisingly no stable emulsion was possible using cyclohexane as oil for particle concentrations in water between 0.01 and 2.0 wt % with complete phase separation occurring within 2 h. For octane, emulsion stability to coalescence increased progressively with particle concentration up to 0.2 wt %. The average droplet size however increased from around 60 μm to 150 μm in this range (Figure 10). It is worth noting that relatively stable emulsions (f_o_ = 0.2 after one month) also exist at 0.3 and 0.5 wt % particles where very large oil drops form (4–6 mm), a feature not possible using surfactants as emulsifier. Densely packed catalyst particles around oil drops can be seen in Figure 10 enabling high stability. Similar trends in behaviour are also found in toluene-in-water emulsions, Figure 11.

## Conclusion

In this work we present a set of heterogeneous Ce–Sn–Pd oxide catalysts that can be used as stabilisers of Pickering emulsions. The catalysts were demonstrated to be very efficient and versatile for Suzuki–Miyaura reactions. The presence of low amounts of dissolved Pd indicates a release/capture mechanism with the synthesised metal oxides acting as pre-catalysts, which slowly release trace amounts of catalytically active Pd into solution. Considering the application of the palladium catalysts in a continuous flow setup, recyclability as well as stability of the catalysts was substantiated and levels of leached Pd in the reaction mixture were determined to be below the critical limit for oral pharmaceuticals. Furthermore, the palladium catalysts proved to be stable for more than 30 h in continuous flow using the so-called plug & play reactor. Since this device can feature multiple HPLC columns, it can also be used for multistep synthesis as planned for the APIs valsartan and sacubitril. In addition, successful implementation of heterogeneous Pd catalysts in Pickering emulsions is a first promising step towards the aimed combination of chemo- and biocatalysis for the continuous formation of valsartan and sacubitril via multistep catalytic cascade reactions.

Finally, it could be shown that the two sets of Pd-containing particles act as sole emulsifiers of various oils in stabilising oil-in-water emulsions. Emulsions stable to coalescence for at least six months can be prepared at low particle concentrations (<0.1 wt %).

Future approaches will concentrate on the applicability of the Pickering emulsions for multistep reactions as well as on the proposed procedure for the synthesis of valsartan and sacubitril.
